# When to Cheat: Modeling Dynamics of Paternity and Promiscuity in Socially Monogamous Prairie Voles (*Microtus ochrogaster*)

**DOI:** 10.3389/fevo.2018.00141

**Published:** 2018-09-19

**Authors:** Marissa A. Rice, Luis F. Restrepo, Alexander G. Ophir

**Affiliations:** Department of Psychology, Cornell University, Ithaca, NY, United States

**Keywords:** mating tactics, reproductive success, mate guarding, alternative reproductive tactics, model simulation

## Abstract

In many socially monogamous species, individuals form long-term pair bonds and males mate guard females. Such behavior is thought to help secure intra-pair fertilizations, the result of intra-pair copulations (IPCs), and ensure paternity. However, socially monogamous males are also often opportunistic and seek additional mating opportunities with other females, leaving their partner unguarded. The success associated with a male’s decision to seek more mates over guarding his partner might be impacted by the activity of other males, specifically the proportion of other males leaving their territories to seek extra-pair copulations (EPCs). The amount of EPC-seeking males can impact the likelihood of a given male encountering an unguarded paired female, but also of being cuckolded (losing IPCs). It remains unclear under which conditions it is optimal to stay and guard or seek EPCs. Using field data from socially monogamous prairie voles *(Microtus ochrogaster)* to generate parameters, we used optimal performance modeling (Monte Carlo simulations) to ask when is it most reproductively advantageous for a bonded male to seek EPCs, despite the risk of losing IPCs. We defined three types of males: exclusive mating bonded males (true residents), non-exclusive mating bonded residents (roving residents), and unpaired males (wanderers). We first modeled the success of an individual male living in a context that incorporated only true and roving residents. We next added wandering males to this model. Finally, we considered the effects of including wandering males and unpaired females in our model. For all contexts, we found that as EPC-seeking in the population increases, the potential reproductive benefit for seeking EPCs increasingly outpaces the rate of cuckolding. In other words, we observe a shift in optimal strategy from true residents to rovers among paired males. Our models also demonstrate that reproductive fitness is likely to remain constant, despite the shift toward obtaining success via EPCs over IPCs. Our results show the dynamic nature of reproductive decision-making, and demonstrate that alternative reproductive decisions yield subtle but important differences despite appearing as balanced strategies.

## INTRODUCTION

Mating dynamics are influenced by many factors that include population density, number of competitors, number of potential mates, and available resources (Shuster and Wade, 2003). Over time, species evolve mating systems that maximize reproductive success given their life history and other ecological constraints (Shuster and Wade, 2003). Even within a species, individuals adopt various strategies to outcompete conspecifics for access to mates (Gross, 1996). Reproductive opportunities in the form of access to receptive females represent a limited resource for males, and as a result males engage in various strategies to maximize these opportunities (Emlen and Oring, 1977). Several behaviors that target ways for males to successfully acquire multiple mates have been described and range in the degree to which they involve male-directed or female-directed interactions (Hull and Rodriguez-Manzo, 2009). Another way males commonly attempt to maximize their own reproductive success at the cost of others is through mate guarding. This behavior is thought to have evolved as a countermeasure to contexts in which costs of female mate acquisition are high and males attempt to usurp other males (i.e., cuckoldry), females readily engage in multiple male mating, or both (Zamudio and Sinervo, 2000). Mate guarding is common across taxa in many species, and is observed in an array of mating systems including polygyny and social monogamy (Møller, 1985; Alberts et al., 1996; Jormalainen, 1998).

A variety of mating systems exist in many different forms across animals. Emlen and Oring (1977) have argued that social monogamy emerges when the ecological and social constraints, and the costs associated with them, are too much to maintain polygynous tactics. Thus, the shift toward social monogamy represents a shift from males attempting to monopolize several females toward monopolizing just one female. Interestingly, polygyny and promiscuity are the most common mating system among mammals, whereas monogamy of any form quite is rare (Kleiman, 1977; Lukas and Clutton-Brock, 2013). This suggests that the constraints that have resulted in mammalian monogamy are uncommon and this shift has only occurred a few times in mammalian evolution, making it all the more interesting to consider the forces that have led to such an outcome in this taxa.

Like all mating systems, social monogamy is rich with complex levels of variation in mating behavior and decisions (Mathews, 2002; Reichard and Boesch, 2003; Kokko and Morrell, 2005). For example, by definition, socially monogamous species seek outside mating opportunities while maintaining social fidelity (Lukas and Clutton-Brock, 2013). Taken together, this framework implies that males (and females) should—at some level—attend to the local and immediate social context to inform their mating decisions (i.e., to engage in monogamy or polygyny).

Although a social bond exists between a socially monogamous male-female pair, on average, both sexes will often engage in mating with individuals other than their pair partner. Several benefits to males mating with multiple females have been proposed, including increasing the quantity of potential successful fertilizations, and maximizing reproductive success through multiple mating (Trivers, 1972, but see Tang-Martinez and Ryder, 2005). Similarly, females mating with multiple males is common (although the reasons for this may or may not be the same as those for males; Yasui, 1997; Tang-Martinez and Ryder, 2005; Blocker and Ophir, 2016). Nevertheless, under the context of females mating with multiple males and the assumption that males might maximize reproductive success through multi-female mating, socially monogamous males are faced with a poignant dilemma: they must weigh the decision to guard a pair partner to ensure paternity and diminish cuckolding, against the decision to leave a partner unguarded to seek extra-pair copulations (EPCs). As a result, a diverse array of behavioral, cognitive, physiological adaptations has evolved to aid in enabling some individuals to navigate this tradeoff. These responses might include responses to sperm competition (Birkhead and Møller, 1993), the ability to time quests for EPCs (Birkhead and Fletcher, 1995), the insertion of copulatory plugs (Ginsberg and Huck, 1989), and sequestering and herding females (Sherman, 1989), to name a few. Nevertheless, an important trade-off that males face between ensuring they fertilize the eggs of one female and attempting to fertilize the eggs of another female can be difficult to optimize. This is because the dynamic and fluid nature of the social environment presumably creates a backdrop on which the factors that define the limits of this trade-off are constantly changing.

Socially monogamous mating systems represent an enormous opportunity to explore the reproductive decision-making that occurs among individuals in a population. Although profoundly complex, the social dynamics within a socially monogamous pair are comparatively simple compared to the number of social interactions that are necessary when more than two individuals comprise a breeding unit, as is the case in other mating systems (polygynous, polyandrous, polygynandrous, etc.). The social context of any mating system is fraught with complexity, but the choice to remain with a partner or to pursue other partners is relatively simple in a socially monogamous mating system in which most individuals engage in some form of social pairing. It is from this perspective that we attempt to model social monogamy and the ways in which the social context potentially shapes the decision to remain a sexually exclusive partner, or to engage in multiple mating. To this end, we base our models on one of the best-understood examples of non-human mammalian social monogamy: the prairie vole (*Microtus ochrogaster*).

As briefly discussed above, individuals must make trade-offs that appear to balance competing strategies when determining the best mating decision. The life history of the prairie vole provides a compelling system for investigating reproductive decisions, as this species has a socially complex and nuanced socially monogamous mating system. Prairie voles exhibit pair bonds between male and female partners, and males engage in mate guarding presumably to maximize their paternity (Solomon et al., 2004). Despite the social arrangement associated with pair bonding, some males and females exhibit multiple-partner mating and/or mating outside the pair bond (Ophir et al., 2008). It is important to note that mixed-paternity litters are common among prairie voles (Solomon et al., 2004; Ophir et al., 2008; Rice and Ophir, Per Obs). Interestingly, males appear to demonstrate a predisposition to forming bonds initially (Blocker and Ophir, 2016), whereas females readily mate with multiple males (Wolff et al., 2002). This evidence is consistent with the idea that males in particular are faced with the important dilemma of determining whether they should mate exclusively with one female or not.

The degree to which a male mate guards, compared to the degree to which he seeks outside mating opportunities, determines the broad categories for prairie vole mating tactics. In the field, males who strongly pair bond and guard both their territory and mate are known as residents (Getz et al., 1993). Yet, some residents appear to engage exclusively in intra-pair copulations (IPCs), whereas other males engage in EPCs or both IPCs and EPCs (Ophir et al., 2008). As such we distinguish between these two sub-types of the recognized “resident” tactic as either “true residents” or “roving residents” (see Ophir, 2017). Operationally, we define true residents as individuals that have formed a pair bond, share a home-range with the pair partner, and for whom all their paternity comes from in-pair fertilizations with that pair partner. Roving residents (or simply rovers), on the other hand, are defined as individuals that have formed a pair bond, share a home-range with the pair partner, and for whom their paternity comes from in-pair and extra-pair fertilizations. Furthermore, some individuals do not form pairs, adding a deeper level of complexity to the prairie vole mating system. These individuals live alone, occupy large home-ranges that are not defended, and intrude frequently into the territories of residents (Getz et al., 1993; Ophir et al., 2008). This tactic is referred to as “wandering” (Getz et al., 1993), and by definition male wanderers can only achieve paternity through EPCs and extra-pair fertilizations.

Traditionally, only the distinction between residents (collectively) and wanderers has been recognized and discussed (Solomon and Jacquot, 2002; Ophir et al., 2008). However, the difference in true resident and roving resident behavior creates an important dynamic in which any one or more of the three tactics might be favored at different moments in time (Okhovat et al., 2015). For instance, the resulting paternity gained by the mix of EPCs and IPCs of rovers potentially sets up an intermediate tactic in which these individuals neither maximize the benefits nor minimize the costs associated with the pure IPC tactic of true residents or the pure EPC tactic of wanderers. Based on the ecological constraints of the prairie vole mating system such as the stability and reproductive advantages of adopting a resident strategy over wandering, this intermediate roving tactic could be selected against (Phelps and Ophir, 2009). Nevertheless the existence and persistence of this intermediate tactic is particularly interesting because success is very likely dependent on the reproductive decisions of other individuals in the population. By leaving a partner unguarded, a rover becomes vulnerable to cuckolding from other rovers and wanderers. Therefore, we predict that roving is only beneficial when the probability of copulating with an unguarded female is relatively high, and the risk of being cuckolded is relatively low. The likelihood of encountering females, either guarded or unguarded, and being cuckolded should depend on whether other males seek EPCs.

We aimed to model the potential fitness payoffs of roving behavior and in doing so we attempt to assess under which social contexts roving behavior should be observed most or least. Some models have addressed this choice of guarding vs. EPC seeking and shown that males mate guard more depending on the degree of female infidelity (e.g., Kokko and Morrell, 2005), others have shown that males mate guard more when there is a male skewed sex ratio and competition increases (e.g., Harts and Kokko, 2013). Here, in our model, the number of males in the population is the same, but the proportion of males engaging in roving behavior fluctuates. By manipulating the proportion of other males in a population that engages in a particular tactic, with only a few simple assumptions that are justified by actual prairie vole behavioral observations, we aim to identify population parameters that are influential in defining the emergence (and success) of roving. Specifically, our model asks: *At what point do reproductive benefits of roving outweigh the costs of potential cuckoldry*? Our goal was to identify the tipping point of rover success [i.e., at what proportion of roving vs. true residents in the population does roving become an advantageous (adaptive) tactic]. We approached this aim by beginning with an overly simple social context, and progressively added basic elements of social complexity. We also considered population size for each of the three progressive conditions we created (see below). In other words, we sought to determine when roving should emerge as a viable reproductive tactic.

## MATERIALS AND METHODS

Our model was designed to assess the optimal decision-making for a hypothetical resident male based on variable social conditions. We began by creating an optimality model that simulated the probability of reproductive success for a single roving male (i.e., the “focal male”). The focal male could achieve reproductive success by mating within the pair bond, outside the pair bond, or both. Our model utilized a Monte Carlo simulation, which allowed us to quantify the ideal outcome of the focal male’s behavior, given various ecological parameters. Our model simulated the reproductive success that a rover could experience when the proportion of true residents and rovers varies. We based the probability of the subject encountering an unguarded female on the home-range size of a resident (true or rover) observed from radio tracking data in semi-natural enclosures (see Ophir et al., 2008). We ran simulations at pair population sizes of 6 (i.e., 6 males and 6 females total) based on Ophir et al. (2008). We also considered the outcome of population size by running simulations with 100, 200, 500, and 1,000 male-female pairs. Population distribution was estimated from Ophir et al. (2008), holding population density constant at 200 voles per ha (but see Getz et al., 1987; Ophir et al., 2008). We varied the population sizes but kept the proportion of animals per unit space constant because social dynamics do not necessarily scale linearly. For each population size, we ran 5,000 simulations for each percentage of roving (from 0 to 100%).

We designed the focal male to rove as our default because our aim was to determine under what social contexts (if any) that roving would ever be a superior tactic to being a true resident. We compared the focal male’s simulated reproductive success to a baseline measure of success typically achieved by true residents. By our definition, a true resident cannot be cuckolded and will achieve all possible reproductive success via IPCs, but he cannot achieve any reproductive success via EPCs. Therefore, if the hypothetical (roving) focal male’s simulated reproductive performance is above the (true resident) baseline, then the decision to rove has a higher probability for maximal reproductive success, indicating that a given male should adopt a roving tactic. However, if the focal male’s simulated reproductive success is less than the baseline, then the decision to rove has a lower probability for maximal reproductive success, indicating that a given male should adopt a true resident tactic (see below for more details).

### Layout of the Mating Field

When constructing our model, we started by creating a general layout of our mating environment where all of our simulations took place. An *N × N* square grid was composed of a number of “tiles” that equaled the product of the dimensions of the enclosure. For example, a 10 × 10 m space would have a grid of 100 m^2^, or 100 tiles. In field experiments using prairie voles in semi-natural enclosures (Ophir et al., 2008), the size of the actual grid was 20 × 30 m, or 600 m^2^ for 6 pairs of animals. The number of tiles in our simulated space for each simulation was held constant at 100 tiles per pair. Thus, a population with 1000 pairs was constructed of a 100,000 tile grid.

The simulations were structured with parameters indicating the location of each tile and a corresponding status for each tile: Empty, Guarded, or Unguarded. Radio telemetry data from Ophir et al. (2008) showed the average home-range size a pair occupied was roughly 40 m^2^. Therefore, we limited an inhabited home-range for a pair to 40 individual tiles in our model. Encounter rates of females for the focal male and every other male utilized a random number generator from 1 to 100,000. Of these 100,000 tiles, hits were ordered as 1 to 40,000. If the focal male generated a number less than or equal to 40,000 at the start of a simulation, then it registered as a successful encounter of a home range. The total tile number decreased by 40 each time a home range was visited, making that home range unavailable thereafter. Thus, a male could encounter a female by landing on any of the 40 tiles constituting a single home range. We acknowledge that this is an oversimplification ofmating behavior, as a male landing a single portion of the territory in nature would not guarantee encountering a female. However, because of the computational constraints of our model, we assume that landing on a tile within a territory will result in an encounter with the female on that territory. And if the female is unguarded on the territory, it will result in a copulation. During a “copulation search” in our simulation (see below), if a given tile on which the focal male explored was “inhabited” by a pair but the resident male of that tile left to seek EPCs (i.e., rove), then that tile was classified as “unguarded.” Alternatively, if the resident male remained within the territory (i.e., a true resident), then the tile was considered inhabited and was classified as “guarded.” If that tile was “uninhabited” by a pair, it was classified as “empty.” The foundation of our simulation was based on probabilities of encountering an unguarded or guarded female to quantify reproductive success in order to observe how social context impacts reproductive success.

### Scoring Scheme for Reproductive Success

Our model was designed to capture the tradeoff between increasing paternity by gaining EPC offspring at the cost of potentially losing IPC offspring. We assumed that by leaving the territory on a foray for additional mating opportunities (thereby leaving the female partner unguarded), a focal male ran the risk of losing IPC offspring due to cuckoldry. Ecologically these may or may not occur simultaneously and we acknowledge that the prospect of finding and achieving an EPC and the threat of being cuckolded are continuous variables in time. We also acknowledge that fertilization of pups within a litter is often attributable to a copulation (or a round of copulations closely linked in time). However, due to the limitations and structure of our Monte Carlo based model, we could only account for mating success of the hypothetical roving focal male in discrete serial events.

**FIGURE 1 |:**
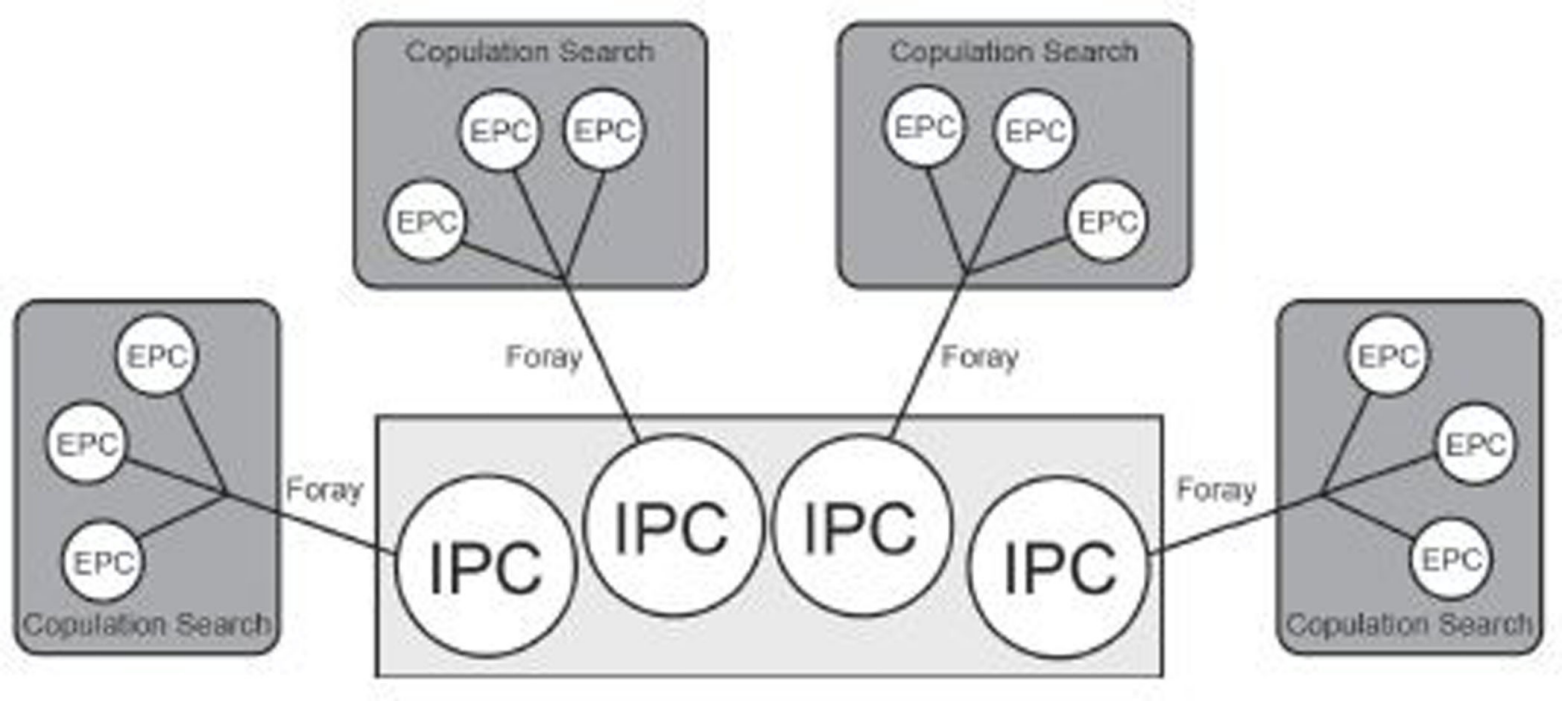
Conceptual diagram of the model portraying the tradeoff between intra-pair copulations (IPC) and extra-pair copulations (EPC). The Monte Carlo method limited us to treating each of the four IPC-EPCs tradeoffs as discrete points in time. In each simulation, the focal male could gain a maximum of 12 EPC offspring (each representing an independent chance), at the potential cost of up to four IPC offspring. A foray refers to each time that the focal male left his territory and risked one IPC for up to three EPCs. Each foray resulted in a copulation search, which refers to the three opportunities to gain up to three EPC offspring.

Furthermore, our model was also based on the premise that the average prairie vole litter size is four (Getz et al., 1993). Because the nature of the Monte Carlo method limited us to using discrete time points, we deconstructed the composition of the litter across time, such that for each IPC pup a male had to lose, he simultaneously gained one opportunity to foray for up to three EPCs, each of which could result in one pup. Thus, each male could gain up to 12 EPC chances that could result in up to 12 offspring, at the potential cost of four IPC offspring (Figure 1). Note that our simulated male only retained an IPC if he was not cuckolded while on a foray.

We use the term ***Foray*** to refer to each time that the focal male left his territory. Each foray resulted in what we refer to as a ***Copulation Search***. A copulation search specifically refers to the three opportunities to gain up to three EPC offspring that a male has on each foray (Figure 1).

For simplicity, we assumed that a successful copulation (IPC or EPC) translated into a successful fertilization. However, we acknowledge that one copulation does not necessarily translate into fertilization. We recognize there are more complex possibilities due to sub-optimal mating events, sperm competition, and physiological conditions that lead to unsuccessful fertilization. For simplicity, we ignore these important sources of variation in fertilization outcomes, and focus on copulation as the key prerequisite of fertilization. As a result, our approximations of fitness rely solely on mating opportunity and the ratios of EPCs vs. IPCs.

On each foray, the focal male risked one IPC for three chances to successfully encounter females, but encountering an unguarded female was not guaranteed. If the focal male successfully encountered an unguarded female during one of the three chances in a copulation search, he gained one EPC. The choice of three EPCs per foray was partially arbitrary, but based on our intention to closely counterbalance the potential reproductive pay-offs and the risk-reward tradeoff of potentially losing an IPC for the chance at acquiring some number of EPCs. Importantly, we wanted to balance the number of potential EPCs with the number of potential IPCs to avoid over- or under-inflating the tradeoff value. Because the chance of IPC was high as long as the focal male did not attempt a foray, the incentive for EPC had to be large, but not guaranteed. Thus, our desired tradeoff of 3:1 should result in an average total fitness value of approximately 3 to 5 pups (following 5,000 simulations), or about the average litter size to ensure biological plausibility.

Taken together, our model created four discrete time-points (forays), where the focal male could gain up to four offspring in each copulation search; three through EPCs and one through IPC. However, the simulated rover was not guaranteed to achieve any EPCs and could lose an IPC if cuckolded by another rover during the copulation search. Therefore, if the focal male never left the territory, then he was guaranteed to not be cuckolded. Using this tactic, the male would forgo all four copulation searches resulting in successful IPCs, but he would achieve no EPCs. If the male always left the territory, the best possible outcome would be 12 offspring from EPCs (see above and Figure 1). On the other hand, a roving male might achieve no EPCs on each of the four forays, and could be cuckolded at each turn, producing a total reproductive success of 0. Intermediate numbers of offspring could also be achieved if the focal male was only cuckolded some of the time and successfully achieved EPCs some of the time. Because each foray represented a single point in time, the focal male’s female partner could not be visited by more than one male; she could only be visited by a cuckolding male, or no male on each turn. In the event that no male visited the partner, the focal male retained an IPC.

Formally, we calculated the number of total copulations by the simulated focal roving male (or reproductive success; R) using the equation: R = E + I − C. Here, E = EPC (or a mating with an unguarded female), I = IPC (or a guaranteed copulation due to successful mate guarding, or no rover intrusion), and C = Cuckold (or the number of times the simulated rover was cuckolded). As detailed above, the total R could vary between 16 (E = 12, I = 4, C = 0) and 0 (E = 0, I = 4, C = 4), and a true resident would yield an R of 4 (E = 0, I = 4, C = 0). Thus, roving can potentially maximize reproductive success only when R > 4. When R = 4, roving and true residency should be equivocal, and roving would be associated with fitness costs (loss of reproductive opportunities) when R < 4.

### Statistical Model for Reproductive Success

As discussed above, each focal male had four chances to either rove (leave the territory) or mate guard (remain at the territory). Each time a focal male chose to rove, he had three chances to gain up to three EPCs. We calculated the focal male’s R for each of those three EPC attempts by producing a value of E between 0 and 3 and a value of C either 0 or 1. The values of E and C were determined via the probability of events. For an *N × N* tile grid with T tiles (*N* × *N* = T), only T−1 tiles were available to be visited by the focal male. Of those total possible tiles to visit, T−1 was divided into three groups: guarded, unguarded, or empty. The number of tiles that were inhabited and unguarded was TU. Thus, TU/(T−1) was the probability of the focal male visiting an unguarded female, if he was roving on the first attempt of the three in a copulation search. The probability on the second attempt was (TU−1)/(T−2). And the probability on the final attempt of the copulation search was (TU−2)/(T−3). Each of the four forays, representing an opportunity to leave the territory to rove, was necessarily treated as independent of each other and therefore the probability of achieving an EPC if the focal male roved was reset to (TU)/(T−1) to begin each search.

The different probabilities for each of the three possible EPCs within a copulation search accounted for the need to exclude the visited tiles during that search. Based on these probabilities, a random number was generated between 1 and the remaining unvisited tiles in that search ([T−1] for the first attempt, [T−2] for the second, and [T−3] for the third). If the number generated was between 1 and the number of remaining unguarded tiles, the focal male was considered to have successfully mated and the value of E increased by one. If any other number was generated, E did not increase. This process repeated two more times for the search, adjusting for the shrinking number of total tiles and unguarded tiles. We reset the number of tiles for the next search and repeated this until all four forays were complete. Similarly, we used the same method to determine C for all roving males, not including the focal male if he was roving. Unlike R, however, the value of C could not be >1.

Recall, the goal of this model was to assess the value of roving compared to mate guarding. Thus, we used a baseline value of R = 4 as a benchmark to compare R of rovers. Our model was designed to quantify a given roving focal male’s reproductive success when the percentage of rovers in the population varied. Thus, we varied the percentage of rovers from 0 to 100% in 10% increments. By varying the proportion of other males that engaged in roving, we were able to assess the threshold at which point a given male would benefit most by adopting a true resident tactic or a roving tactic given the average tactic of other males in the population.

We wanted to consider more complexity in our populations in the second iteration of the model (Condition 2) to better characterize the mating dynamics of prairie voles in the wild. To this end, we created a model as described above that also included male wanderers (i.e., males that remain unpaired and only acquire mates with unguarded females). In natural populations, the occurrence of wanderers varies from 10 to 40% (Thomas and Birney, 1979; Getz et al., 1993; Solomon and Jacquot, 2002; Ophir et al., 2008). We ran the entire model (5,000 simulations per roving percentage, 0 to 100%) with the addition of 10, 20, 30, or 40% wandering males in our Condition 2 simulations. The mechanics of the wandering males and their impact on the focal male used the same method to determine C for all roving males used in Condition 1 simulations. We predicted that adding wandering males would reduce R for the roving tactic because it would create greater male-male competition increasing the costs of leaving females alone.

In a final iteration of the model (Condition 3), we modified Condition 2 to account for an additional important social factor: availability of females. Typically, prairie vole sex ratios are relatively balanced overall (Getz et al., 1981). By adding additional male wanderers to the simulations in Condition 2, we created an unbalanced and male biased sex ratio that could profoundly impact the degree of competition, importance of mate guarding, and the ultimate R that any given male might achieve. Thus, in Condition 3 we simulated a balanced sex ratio that incorporated both resident and wandering males to avoid having an unnaturally skewed male to female sex ratio. To this end, we added unpaired and unguarded females to correspond to every male wanderer introduced in Condition 2. The mechanics of the added unpaired females were the same as the unguarded females in Conditions 1 and 2. As before, we ran the entire model (5,000 simulations per roving percentage, 0 to 100%) with the addition of 10, 20, 30, or 40% wandering males and the corresponding number of unpaired females in Condition 3 simulations. We predicted that adding unpaired females would restore any lost value of R observed in Condition 2 to levels comparable to those seen in Condition 1.

## RESULTS

**FIGURE 2 |:**
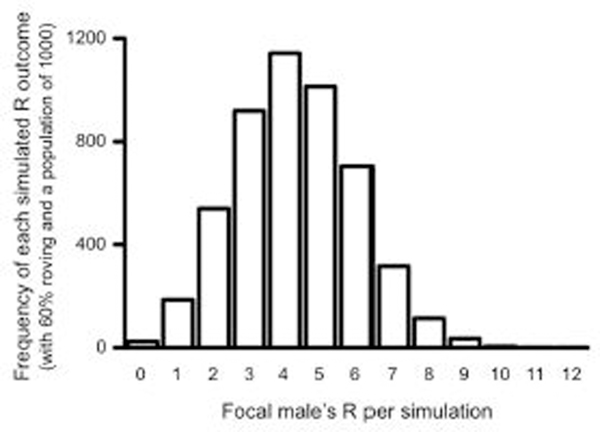
Histogram of total reproductive success (R) outcomes for the simulated roving focal male after 5000 simulations. The histogram represents the simulation results for which 60% of the males in the population roved, and the pair population was 1,000.

As stated above, each simulation was run 5,000 times at each percentage of roving (from 0 to 100%) in the population (6, 100, 200, 500, and 1,000 male-female pairs) to ensure that our measures of reproductive success were normally distributed. To confirm this, a histogram was generated for each roving percentage in the population. As expected, all outcomes of the 5,000 simulations were normally distributed at each percentage of roving in the population. Figure 2 presents the results of one such simulation at 60% roving with a population of 1,000 males and females as an example. All results are reported as the average reproductive success values. Because all pair populations exhibited the same patterns, we primarily focus on the simulations of the largest pair population size (1,000 pairs) below to eliminate redundancy. Nevertheless, results from other population simulation data are reported in Supplementary Material.

### Condition 1

**FIGURE 3 |:**
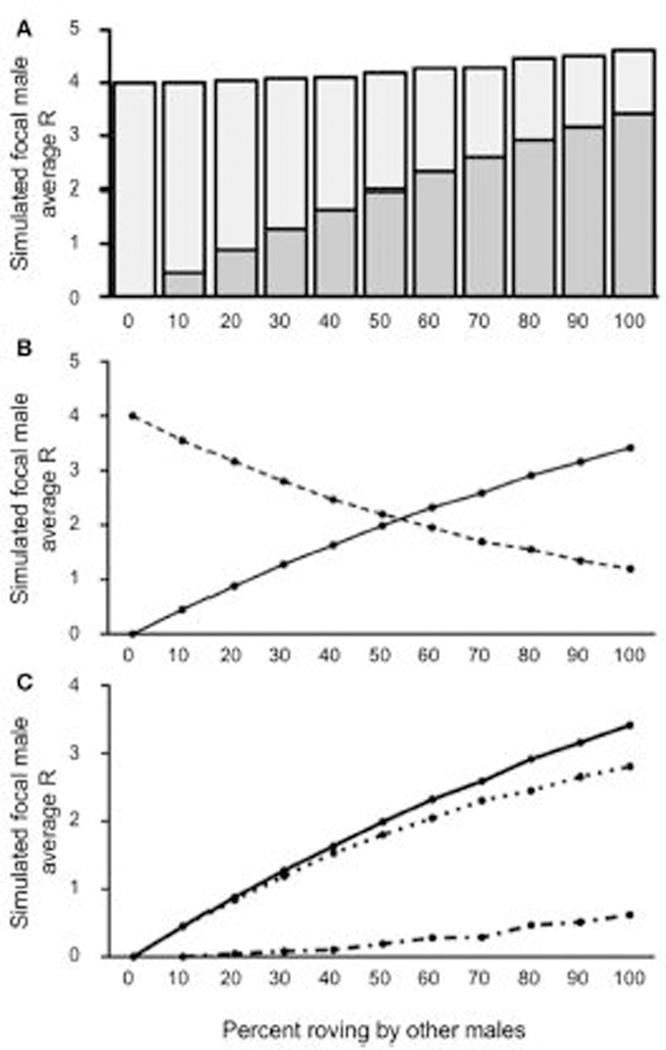
Reproductive success for simulated roving focal male In Condition 1 at pair population 1,000. **(A)** Mean reproductive success (R) obtained by the simulated roving focal male at each percentage of roving In the population, split between R gained via extra-pair copulations (EPC; dark gray) and Intra-pair copulations (IPC; light gray). **(B)** Average R gained via EPCs (solid line) and IPCs (dashed line) by the simulated roving focal male as the percentage of roving In the population increases. **(C)** Average R gained via EPCs ($\bar{E}$, solid line), average R lost via cuckolding ($\bar{C}$, dotted line), and total change in R (ΔR, dash-dotted line) for the simulated roving focal male as the percentage of roving In the population increases.

Our first model attempted to over-simplistically characterize the reproductive success of rovers given other male tactics in the population. Figure 3A presents the value for R, represented both as IPCs and EPCs, across the percentages of roving in the population. We used one-sample *t*-tests to compare the simulated focal male’s R to the expected baseline of a true resident tactic (R = 4) for each column. *T*-test significance thresholds were adjusted for multiple comparisons using the False Discovery Rate (FDR) correction (Benjamini and Hochberg, 1995). Our results indicate that the focal male gained significantly more R than baseline [all *t*’s_(4999)_ > 2.62; *p <* 0.0104] in all but the 0–10% roving conditions. When no males in the population were roving (0% roving), the simulated focal male gained no EPCs, presumably because all the females in the population were always guarded. Also, when no males in the population roved, the simulated focal male retained all IPCs because no other males were seeking EPCs. As a result of these constraints, the simulated (roving) male could not achieve a reproductive success value other than 4 at 0% roving in the population, producing an outcome of R = 4 without variance. Although all the outcomes for R for the focal male at 10% roving were greater than baseline, the increase in the actual R gained was qualitatively marginal, and non-significant [*t*_(4,999)_ = 0.08; *p =* 0.9347]. At the other extreme, when 100% of the males in the population roved, the simulated focal male achieved the greatest R (4.614). This represents a 13.3% increase over the expected true resident R of 4. Interestingly, the composition of R was drastically different (more EPCs than IPCs) as the percentage of roving increased in the population (see Figure 3B).

When 50% or less of males in the population roved, the majority of the simulated focal male’s reproductive success resulted from IPCs (2.19–4.00) rather than EPCs (0.00–1.99). In contrast, when more than 50% of males in the population roved, most of the simulated focal male’s reproductive success came from EPCs (2.32–3.41) rather than from IPCs (1.19–1.95). The point at which EPC’s contributed more to total R than IPCs occurred around 56% roving in the population. Despite this trade-off, the total R from both IPCs and EPCs was relatively constant, but slightly increased (see above). As a result of this relationship, the overall increase in reproductive success (ΔR) increased as a function of the relationship between the average acquired EPCs $\left(\bar{E}\right)$, and the average amount of cuckolding $\left(\bar{C}\right)$ (Figure 3C). Our model showed that when male roving was uncommon in the population, the $\bar{E}$ and $\bar{C}$ for the simulated focal male increased at similar rates. However, as male roving in the population became more common (i.e., higher percent of male roving), $\bar{E}$ began to outpace $\bar{C}$. This result indicates that males benefit by adopting a roving tactic most when the proportion of other roving males in the population is relatively high.

### Condition 2

We modified the original model to incorporate the influence of wandering males (at different proportions in the population) in a second iteration of our model. Using this framework, we created simulations for wanderers at four proportions (10, 20, 30, and 40% wanderers) and we report the results from the lowest (10%) and highest (40%) proportion of wanderers here; the model results for wandering at 20 and 30% are included in Supplementary Material. We predicted that increasing the presence of wanderers would decrease the simulated focal male’s R due to the increased competition for unguarded females.

**FIGURE 4 |:**
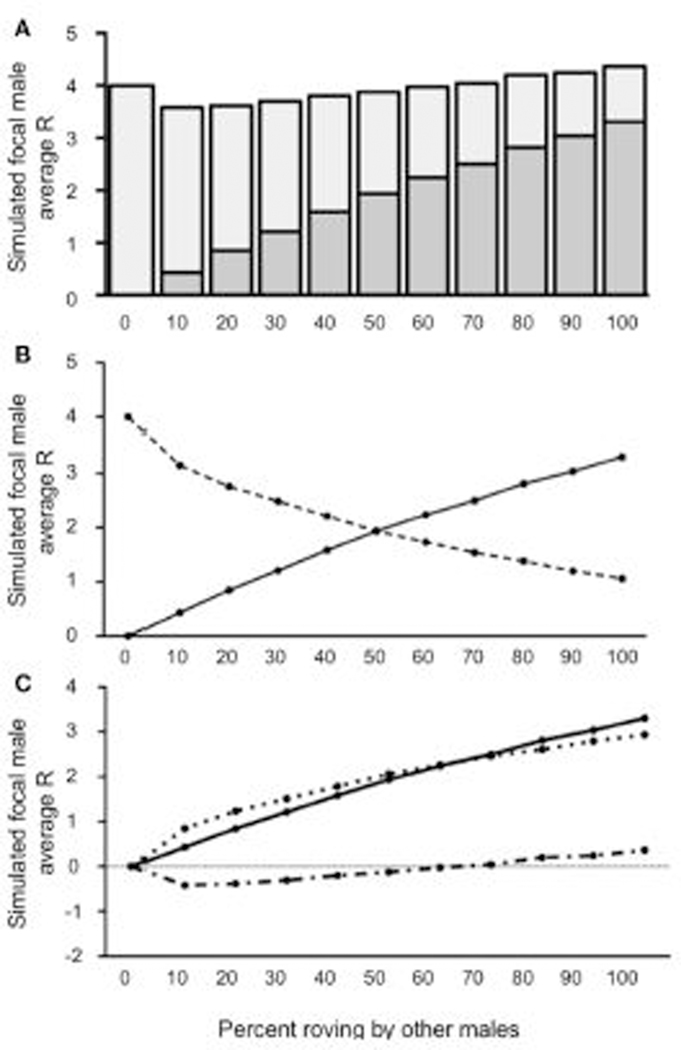
Reproductive success for simulated roving focal male in Condition 2 at pair population 1,000 with 10% wandering. **(A)** Mean reproductive success (R) obtained by the simulated roving focal male at each percentage of roving in the population, split between R gained via extra-pair copulations (EPC; dark gray) and intra-pair copulations (IPC; light gray). **(B)** Average R gained via EPCs (solid line) and IPCs (dashed line) by the simulated roving focal male as the percentage of roving in the population increases. **(C)** Average R gained via EPCs ($\bar{E}$, solid line), average R lost via cuckolding ($\bar{C}$, dotted line), and total change in R (ΔR, dash-dotted line) for the simulated roving focal male as the percentage of roving in the population increases.

Like in condition 1, our model demonstrated that the simulated focal male’s R varied as a function of the proportion of rovers in the population when 10% of the male population adopted a wandering tactic (a relatively low incidence of wandering) (see Figure 4A). Specifically, the optimal tactic was to be a true resident when there was a low incidence of wandering in the population and roving was uncommon. Adopting a roving tactic was beneficial, however, when the proportion of other rovers increased. The simulated focal male’s R was <4 [all *t*’S_(4,999)_ ≥ 5.529; all *p’s* ≤ 0.0001] when 50% or fewer of the males in the population roved, indicating that roving when most males are either wanderers or true residents does not benefit reproductive success. When 60 and 70% of the males in a population roved in the presence of 10% wanderers, the simulated roving male’s R was equal to the baseline true resident reproductive success [R = 4; *t*_(4,999)_ = 1.019; *p =* 0.308 for 60%; *t*_(4999)_ = 1.733; *p =* 0.0831 for 70%]. Notably, when roving in the population in the presence of 10% wanderers increased above 70%, the simulated roving male began to accumulate reproductive success that was greater than the true resident payoff of 4 [all *t*’s_(4999)_ ≥ 8.157; all *p’s* ≤ 0.0001]. Also like in Condition 1, the majority of copulations switching from IPCs to EPCs occurred between 50 and 60% roving (Figure 4B). However, Condition 2 differed from Condition 1 with respect to the relationship between $\bar{E}$ and $\bar{C}$ (Figure 4C). Specifically, $\bar{E}$ and $\bar{C}$ was greater than $\bar{E}$ for roving population percentages below 60%, resulting in a negative ΔR. Initially the rate of $\bar{C}$ outpaced $\bar{E}$, but plateaued as the roving population reached 60%. Still, $\bar{E}$ was smaller than $\bar{C}$ when roving was relatively uncommon among males in the population. However, $\bar{E}$ steadily increased as the percentage roving in the population increased. Interestingly, $\bar{E}$ overtook $\bar{C}$ as roving became more common in the population, indicating that roving would be beneficial for a given male (positive ΔR) when roving in the presence of a small proportion of wanderers (10%) becomes increasingly common.

**FIGURE 5 |:**
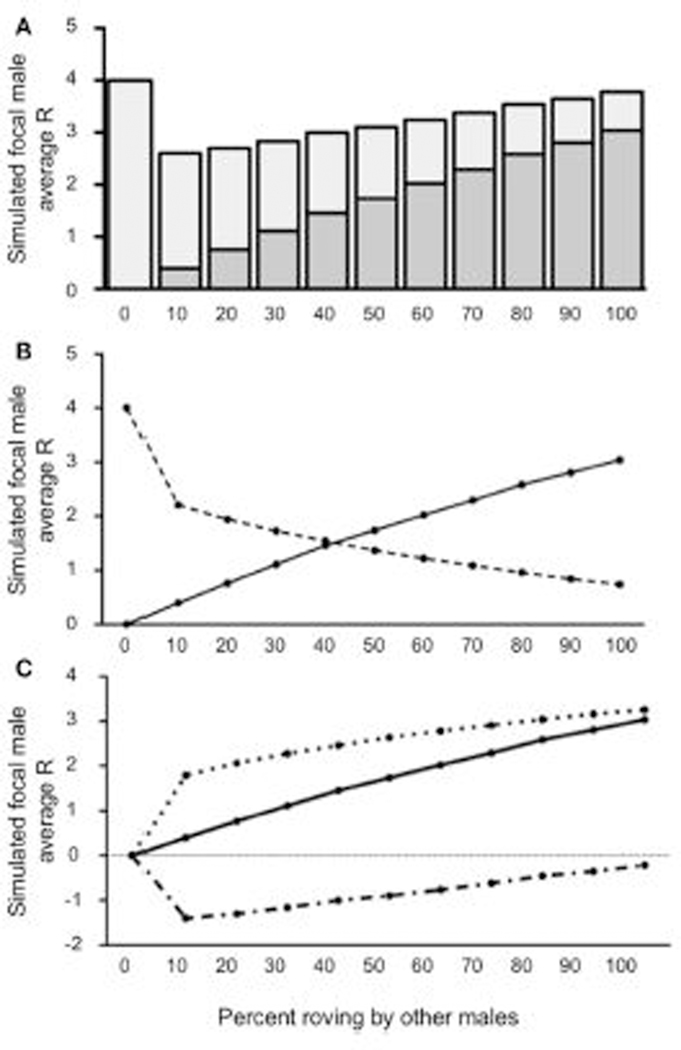
Reproductive success for simulated roving focal male in Condition 2 at pair population 1,000 with 40% wandering. **(A)** Mean reproductive success (R) obtained by the simulated roving focal male at each percentage of roving in the population, split between R gained via extra-pair copulations (EPC; dark gray) and intra-pair copulations (IPC; light gray). **(B)** Average R gained via EPCs (solid line) and IPCs (dashed line) by the simulated roving focal male as the percentage of roving in the population increases. **(C)** Average R gained via EPCs ($\bar{E}$, solid line), average R lost via cuckolding ($\bar{C}$, dotted line), and total change in R (ΔR, dash-dotted line) for the simulated roving focal male as the percentage of roving in the population increases.

The impact of wandering was most notable when the proportion of wanderers in the population was high. Indeed, when wandering was relatively common (40% wanderers in the population), the simulated focal male achieved less reproductive success than baseline (true residents, R = 4) regardless of the proportion of other rovers in the population above 0% [all *t*’s_(4999)_ ≥ 9.441; all *p’s* ≤ 0.0001]. The one exception to this was when no roving (0%) occurred and the simulated focal male’s R was always 4, identical to that of a true resident (Figure 5A). In other words, when wandering was common, there appeared to be no reproductive benefit to roving, and there was usually a reproductive cost. Furthermore, the reproductive costs associated with roving were greatest when the proportion of roving among other males in the population was between 0% and 50%. The point at which IPCs contributed to R less than EPCs occurred much earlier in this simulated scenario than we found in Condition 1 or when wanderers were relatively uncommon (10% wanderers in the population). In this case, EPCs began to account for the majority of the simulated focal male’s R when 40% of the population roved (Figure 5B). Moreover, C increased rapidly throughout the simulations (Figure 5C), such that $\bar{C}$ was very large when roving was relatively rare (i.e., low percentages of roving) and continued to increase steadily as more males in the population began to rove. The $\bar{E}$ also increased as the proportion of rovers increased in the population, thereby leaving more and more females unguarded. The ΔR slowly increased as $\bar{E}$ also increased, but $\bar{E}$ never surpassed $\bar{C}$ in this scenario and ΔR therefore remained negative. Taken together, the results from this model showed that when wandering is common (40% wanderers in the population) the optimal reproductive tactic is to adopt true residency, regardless of the roving percentages in the population.

### Condition 3

**FIGURE 6 |:**
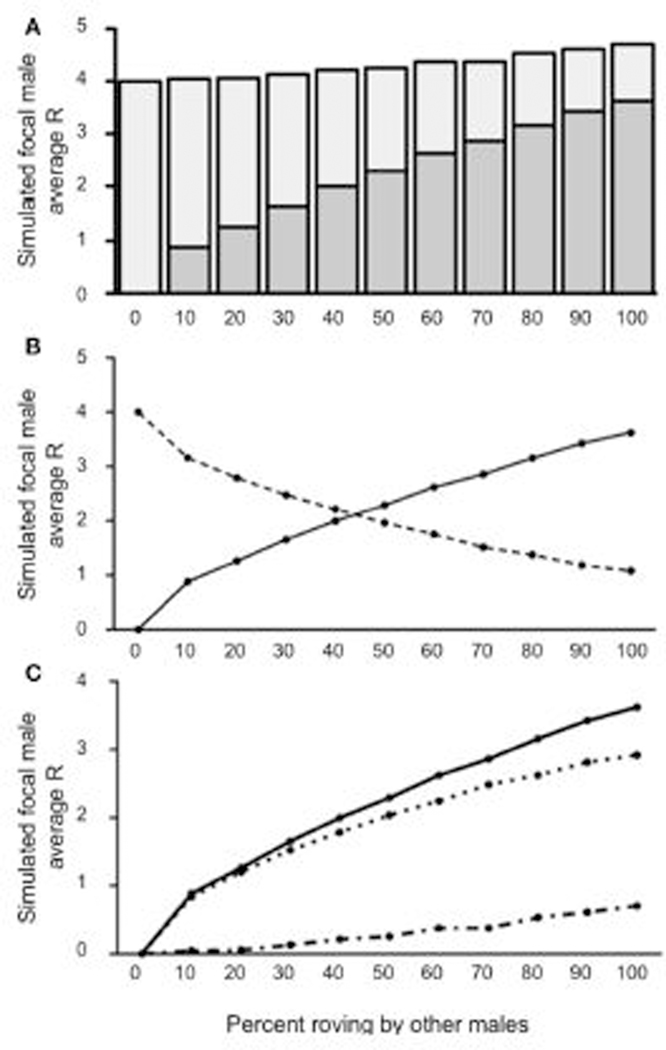
Reproductive success for simulated roving focal male in Condition 3 at pair population 1,000 with 10% wandering and unpaired females. **(A)** Mean reproductive success (R) obtained by the simulated roving focal male at each percentage of roving in the population, split between R gained via extra-pair copulations (EPC; dark gray) and intra-pair copulations (IPC; light gray). **(B)** Average R gained via EPCs (solid line) and IPCs (dashed line) by the simulated roving focal male as the percentage of roving in the population increases. (**C**) Average R gained via EPCs ($\bar{E}$, solid line), average R lost via cuckolding ($\bar{C}$, dotted line), and total change in R (ΔR, dash-dotted line) for the simulated roving focal male as the percentage of roving in the population increases.

In a final set of simulations we incorporated the social contexts discussed in Conditions 1 and 2, again with a focus on accounting for low (10%) and high (40%) rates of wandering in the population, but now adding unpaired females to represent a more equitable sex ratio in the population. Under this context, we found that at low levels of wandering (10% wanderers in the population), the decision to rove was a successful tactic for the simulated focal male, regardless of the proportion of roving by other males in the population from 10 to 100% [all *t’*s_(4,999)_ ≥ 2.799, all *p*’s ≤ 0.0051; see Figure 6A]. As we observed before, when no other males roved (0% rovers in the population) the simulated focal male always achieved the same reproductive fitness if he roved as he would if he adopted true residency (where R = 4). Despite these statistical differences, the simulated focal male’s R was qualitatively only marginally better than the true resident baseline (R = 4) when the proportion of other males in the population that roved was 50% or less. Specifically, when 50% of the males in the population roved R = 4.25 for the simulated focal male, representing a small-scale fitness advantage, but one that could be functionally important over time. Moreover, as the proportion of roving by other males in the population increased beyond 50%, the focal male’s R also increased. When the proportion of other males that roved reached 100%, the simulated focal male’s reproductive success peaked at R = 4.70. Notably, the point at which the simulated focal male’s IPCs contributed less to R than his EPCs occurred when 50% or more of the other males in the population roved (Figure 6B). This total positive change in R was best observable by the result indicating that $\bar{E}$ began to outpace $\bar{C}$ (Figure 6C). These results demonstrate that the addition of unpaired females at low levels of wandering (10%) produced very similar outcomes as Condition 1 and seemed to restore an overall balance between which tactic (roving or true residency) resulted in the maximal reproductive pay-off for the simulated focal male depending on the proportion of other males that decided to rove.

**FIGURE 7 |:**
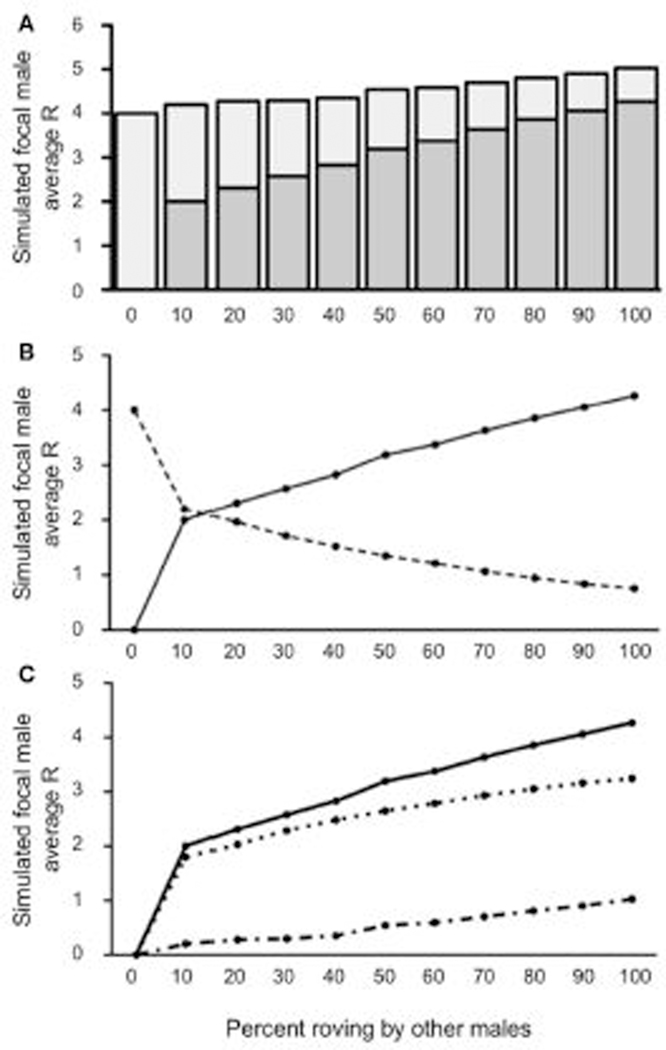
Reproductive success for simulated roving focal male in Condition 3 at pair population 1,000 with 40% wandering and unpaired females. **(A)** Mean reproductive success (R) obtained by the simulated roving focal male at each percentage of roving in the population, split between R gained via extra-pair copulations (EPC; dark gray) and intra-pair copulations (IPC; light gray), **(B)** Average R gained via EPCs (solid line) and IPCs (dashed line) by the simulated roving focal male as the percentage of roving in the population increases. **(C)** Average R gained via EPCs ($\bar{E}$, solid line), average R lost via cuckolding ($\bar{C}$, dotted line), and total change in R (ΔR, dash-dotted line) for the simulated roving focal male as the percentage of roving in the population increases.

High levels of wandering (40%) in the presence of a balanced sex ratio greatly increased the reproductive success of the simulated focal male (Figure 7A). Just like when wandering was uncommon (10%, see immediately above), the simulated focal male’s R was significantly >4 so long as at least some other males in the population also roved [10–100%; all *t’s*_(4,999)_ ≥ 8.719; all *p’s* < 0.0001]. The increase in R was more pronounced when the proportion of other roving males increased. For example, when 100% of other males in the population were rovers, R = 5.02 for the simulated focal male. Not surprisingly, it was under this context where our model predicted the highest rates of EPCs by the simulated focal male than in any other condition for which we ran simulations. Indeed, the model indicated that when wandering is high and a balanced sex ratio provided some proportion of unpaired females, the focal male lost more IPCs on average, but he also gained more reproductive success via EPCs to compensate and surpass those losses. Notably, this shift in the source of reproductive success (IPCs to EPCs) occurred at the earliest point for all conditions and scenarios we modeled—occurring between 10 and 20% of other males’ roving in the population (Figure 7B). Because the simulated focal male was able to compensate for the loss of IPCs with additional EPCs, the rate of $\bar{E}$ outpaced $\bar{C}$ throughout the simulation resulting in a consistently positive ΔR (Figure 7C). Taken together, our model predicted that at high levels of wandering (40%) and in the presence of additional females in the population, the simulated focal male has a high probability of benefiting from adopting a roving tactic, and reproductive success should continually increase as the rate of roving in the population also increases.

## DISCUSSION

Taken together, our models demonstrated that the decision to rove is strongly dependent on the social environment and the degree to which other males have adopted roving and/or wandering alternative tactics. In general, the more common roving becomes in a population, the greater the average fitness payoff is likely to be if a given male decides to rove in kind. The point at which the pay-off to switch to roving from true residency tends to occur is when half or more of the males in the population rove. Whereas the presence of wanderers in the population increased the net reproductive pay-off of guarding partners and the ultimate value of adopting true residency, the availability of females in the population had the opposite effect. The consistent pattern across all of our simulations showing that reproductive success (R) for roving increases as the proportion of roving by other males increases is noteworthy. This is because although rates of cuckolding increased as male roving became more common (representing a potential fitness cost for the simulated focal male), the simulated focal male’s reproductive success from EPCs also increased, and at a rate that outpaced cuckolding.

### A Putative Context in Which an Intermediate Strategy Might Evolve

Most mating systems that have alternative tactics usually have two dominant forms. Frequently this is observed as the “territorial”/“sneaker” dynamic (Oliveira et al., 2008). The rarity for more than two tactics to occur in a system is often explained by disruptive selection operating against the intermediate tactics. This would certainly be a more powerful explanation when prominent morphological differences (particularly those that are dictated by developmental pathways) are associated with the alternative tactics. Our model offers potential insight into the ways in which behaviorally based intermediate tactics might evolve. Specifically, our model supports the idea that the social context contributes to the costs and benefits of engaging in the intermediate mating tactic of roving. The behaviorally flexible nature of the tactic in this system is an underlying necessity for this intermediate tactic to be successful. As expected, some population parameters of our model limited the reproductive payoffs associated with roving, resulting in this tactic being detrimental. For example, when wandering was relatively common (40% wanderers in the population) and approximated the rate of wandering observed in some field studies (Thomas and Birney, 1979; Getz et al., 1993), roving was associated with a loss in fitness relative to remaining exclusive and guarding a partner. This was particularly true when the sex ratio was skewed toward more males (i.e., a male biased sex ratio; Condition 2). Similarly, roving tended to be more costly than true residency with a male-biased sex ratio, even when wandering was relatively rare (10% wanderers in the population) and approached a rate that is just under what has been observed in the wild (Ophir et al., 2008). However, under this scenario the presumptive cost of roving was relaxed as roving in the population among other males increased beyond 60%. Still, under a male-biased context with the threat of cuckoldry from other resident males in the population and wanderers present, the fitness costs to roving should limit the frequency of this tactic. Despite these outcomes from our model (particularly under Condition 2), and the theoretical logic that underlies them, roving persists in free-living populations of prairie voles (Solomon et al., 2004; Ophir et al., 2008) suggesting that the conditions that lead to selection against roving must be uncommon, or understudied. Indeed a context with a more equitable sex ratio (like those modeled in Condition 3) is probably more representative of the natural contexts in which the male reproductive decision-making takes place (see below). Importantly, our model demonstrated that the decision to rove should be flexible and contingent on the social context. It also demonstrated that several social contexts not only permitted the viability of roving, but in many cases that roving was associated with a net benefit over true residency. Indeed, our model indicates that although selection should operate against roving among male prairie voles under some contexts, there are several other contexts in which this “intermediate” tactic can be quite successful.

### Balanced Outcomes of Reproductive Success

In a few cases, our model indicated that the reproductive success of the simulated focal male when roving was equivalent to that of true residents. This was true in Condition 1 at 10% roving, and Condition 2 at 60 and 70% roving, for example. Although this outcome did not account for the majority of scenarios we modeled, it did indicate that under some cases the pay-offs of roving would be no better than to mate guard as a true resident. Under these conditions, there are some additional considerations that could bias males to decide to either rove or remain exclusive. For example, our model did not account for the energetic costs associated with guarding or with roving and these would certainly impact the decision of which tactic to adopt. Venturing outside the safety of the territory and home range to engage in EPCs should increase predatory risks and the potential for aggressive encounters with other males, which might bias males to avoid roving when all else is equal. On the other hand, our model did not account for conditions where males could seek EPCs with minimal cost by roving only after intra-pair fertility was secured. Some examples include when the female is past receptivity, pregnant, or caring for pups. Perhaps the incidences of roving and EPCs would be higher than predicted in natural populations when considering these factors. Another consideration is that roving might benefit males if increasing genetic diversity (Hasselquist et al., 1996) or avoiding the potential costs associated with placing “all eggs in one basket” (from predation or disease for example, Krokene et al., 1998) are important factors in a system. Surely factors like these and others that our model did not consider could bias males’ reproductive decisions. Importantly, second order fitness payoffs should be considered if/when the reproductive payoffs are equivalent. Wolff and Macdonald (2004) argued that when all else is reproductively equal, males that remain at the nest and invest in offspring will ultimately outperform males that invest less in their offspring. Engaging in a tactic that enables more paternal care is more likely to produce offspring with greater survivability, if for no other reason other than spending more time at the nest increases the probability that pups will be defended, groomed, thermoregulated, etc. Thus, even if the reproductive costs of successfully fertilizing offspring for a partner or stranger are equivalent for rovers and true residents, the investment in own offspring could provide greater fitness payoffs for true residency and outweigh the payoffs of roving. In support of this notion, pups raised without fathers demonstrate behavioral and neural phenotypes that could produce offspring that are less prepared for success as adults (Wang and Novak, 1992, 1994; Ahern and Young, 2009; Prounis et al., 2015).

For every iteration of our model, we found a point where reproductive success from EPCs began to contribute more to the simulated focal male’s total R than the reproductive success gained from IPCs. This is an interesting finding considering that previous models have found that males should increase mate guarding as male competition increase in order to preserve paternity (Harts and Kokko, 2013). Our model brings to light an alternative tactic to maximize paternity in the face of increased male competition; to shift to predominantly seeking EPCs. Despite the fact that our simulated focal males gained paternity from EPCs, they also suffered reproductive costs from cuckolding, which limited the average total success that our simulated focal males stood to gain as rovers. This tradeoff is seen in many species, particularly in synchronous breeding conditions, where males gain much of their EPCs when their mate is also fertile and most susceptible to cuckolding (Stutchbury and Morton, 1995; Grunst et al., 2017). If in our simulation $\bar{E}$ did not outpace $\bar{C}$, then a true resident strategy would be more advantageous and the costs of roving would not outweigh the benefits. Thus, the switch in composition of R represents a potential switch for which rovers may benefit more by primarily pursuing EPCs. In other words, this switch-point captures the trade-off that rovers face: having half of copulations outside a pair ensures some paternity in the event of nest destruction, while potentially raising the young of another male is disadvantageous.

### Optimizing Strategy or Merely Getting Lucky

Our model considered the reproductive pay-offs of roving. However, it is unclear if roving is the default tactic in nature. Mixed paternity has been observed in field experiments (Solomon et al., 2004; Ophir et al., 2008), providing strong evidence that prairie voles commonly adopt roving and/or wandering strategies. Even if residency (broadly defined) is the preferred or default tactic among prairie voles, no study of which we are aware has attempted to characterize the frequency of roving residents to true residents, or whether one has a clear reproductive advantage over the other. The current study attempts to predict the contexts in which roving and true residency should be observed, the frequencies at which they should be found, and estimate the potential reproductive pay-offs of the two tactics when they are found. Still, little is known about the individual decision to pursue each tactic. The information that an animal relies upon to best inform mating decisions is often incomplete. It is for this reason that assessment of the social context (however complete or flawed it might be) should serve as a valuable and relatively easily acquired source of information on which inferences about the population could be based. Social information could, thereby, serve as grounds on which reproductive decisions could be based (Jarrige et al., 2015).

Similarly, mating decisions can result in behavioral reinforcement that perpetuates previous decisions. For example, if a male successfully mates with a female other than his partner (regardless of if she is unpaired, or paired and unguarded), the decision to seek EPCs in the future should presumably be reinforced. Likewise, if a resident male encounters and expels several EPC-seeking intruders while mate guarding, he is likely to continue mate guarding. Conversely, regularly encountering other EPC-seeking males while searching for EPCs might indicate to a given male that the risk of being cuckolded is high, and encourage that male to return to his territory. Although mate guarding could plausibly offset the risk of cuckoldry, our model indicates that males should continue seeking EPCs when other EPC-seeking males are common in order to offset the high risk of cuckoldry. Similarly, low rates of intrusions might inform a mate guarding resident male that risk of cuckoldry is low, in turn increasing the probability that that male will search for EPC opportunities.

Whatever the mechanisms that account for the decisions males make, we believe that the social environment offers a rich set of information that individual animals can and should use to assess the costs and benefits of adopting a particular tactic at a given moment in time. For example, in the lekking lesser wax moth, *Achroia grisella,* males change mating behavior depending on the perception of other male competition (Jarrige et al., 2015). When the experimenters included another male observer during mating (perception of increased competition), the focal male mated the female more frequently allocating more sperm than when no observer was present (Jarrige et al., 2015). Additionally, a similar conceptual approach, relying on information in the social environment, has been used to review extra-pair paternity in birds (Maldonado-Chaparro et al., 2018). Maldonado-Chaparro et al. (2018) argue that the local social environment (information about a pair partner) and the extended social environment (individuals in the population) contribute to individual mating decisions.

Finally, we believe that effort aimed at assessing the behavioral mechanisms and the modes by which social information is gathered and processed is tremendously important if we are to ever fully begin to understand the cognitive ecology that sub-serves reproductive decision-making. Ultimately, the extent to which mating tactics are flexible and individuals are sensitive to social information will serve as one of the pillars upon which the foundation of understanding the dynamics of mating systems is broadly built. It is our hope that the current study highlights the potential importance of social information on reproductive decision-making, the adoption of mating tactics, and the mating systems that ultimately emerge from them.

## Supplementary Material

supplementary material - Table 1
