# Supplementary material for: When to Cheat: Modeling Dynamics of Paternity and Promiscuity in Socially Monogamous Prairie Voles (Microtus ochrogaster)
Source: Front Ecol Evol. Author manuscript; Available in PMC 2021 Jul 1. (PMC8248529; doi:10.3389/fevo.2018.00141)
Supplement: supplementary material - Table 1 [file NIHMS1609269-supplement-supplementary_material_-_Table_1.pdf]

Condition 1. Reproductive success for simulated roving focal male in Condition 1 at pair population 6 at each percentage of roving by other males in the population.

Pair Population 6	0%	16.67%	33.3%	50%	66.67%	83.3%	100%
**Average Total R**	4.00	4.00	3.93	3.94	3.98	4.06	4.17
**Average IPC**	4.00	4.00	3.13	2.48	1.95	1.55	1.19
**Average EPC**	0.00	0.00	0.80	1.46	2.03	2.51	2.98
**Average C**	0.00	0.00	0.86	1.52	2.04	2.45	2.81
**ΔR**	0.00	0.00	-0.06	-0.05	-0.01	0.06	0.17

Condition 1. Reproductive success (R) for simulated roving focal male in Condition 1 at pair population 100 at each percentage of roving by other males in the population.

Pair Population 100	0%	10%	20%	30%	40%	50%	60%	70%	80%	90%	100%
**Average Total R**	4.00	4.01	3.99	4.09	4.07	4.17	4.24	4.32	4.38	4.47	4.59
**Average IPC**	4.00	3.58	3.15	2.83	2.48	2.22	1.94	1.71	1.54	1.36	1.20
**Average EPC**	0.00	0.43	0.84	1.26	1.59	1.95	2.30	2.61	2.84	3.11	3.39
**Average C**	0.00	0.42	0.85	1.16	1.51	1.77	2.06	2.29	2.46	2.63	2.79
**ΔR**	0.00	-0.01	-0.01	0.09	0.07	0.17	0.24	0.32	0.38	0.47	0.59

Condition 1. Reproductive success for simulated roving focal male in Condition 1 at pair population 200 at each percentage of roving by other males in the population.

Pair Population 200	0%	10%	20%	30%	40%	50%	60%	70%	80%	90%	100%
**Average Total R**	4.00	3.99	4.02	4.10	4.10	4.18	4.22	4.34	4.43	4.47	4.56
**Average IPC**	4.00	3.57	3.16	2.81	2.49	2.22	1.94	1.73	1.55	1.34	1.21
**Average EPC**	0.00	0.42	0.86	1.29	1.61	1.96	2.28	2.61	2.88	3.13	3.35
**Average C**	0.00	0.42	0.84	1.19	1.51	1.77	2.05	2.26	2.45	2.65	2.78
**ΔR**	0.00	-0.01	0.02	0.10	0.10	0.18	0.22	0.34	0.43	0.47	0.56

Condition 1. Reproductive success for simulated roving focal male in Condition 1 at pair population 300 at each percentage of roving by other males in the population.

Pair Population 300	0%	10%	20%	30%	40%	50%	60%	70%	80%	90%	100%
**Average Total R**	4.00	3.99	4.04	4.04	4.10	4.18	4.27	4.36	4.39	4.49	4.58
**Average IPC**	4.00	3.56	3.17	2.79	2.46	2.21	1.95	1.74	1.52	1.36	1.20
**Average EPC**	0.00	0.43	0.87	1.25	1.64	1.97	2.32	2.62	2.87	3.13	3.38
**Average C**	0.00	0.44	0.83	1.20	1.54	1.79	2.04	2.25	2.48	2.64	2.79
**ΔR**	0.00	-0.01	0.04	0.04	0.10	0.18	0.27	0.36	0.39	0.49	0.58

Condition 1. Reproductive success for simulated roving focal male in Condition 1 at pair population 400 at each percentage of roving by other males in the population.

Pair Population 400	0%	10%	20%	30%	40%	50%	60%	70%	80%	90%	100%
**Average Total R**	4.00	4.01	4.01	4.13	4.11	4.19	4.23	4.32	4.44	4.48	4.57
**Average IPC**	4.00	3.56	3.14	2.82	2.46	2.20	1.94	1.73	1.53	1.37	1.18
**Average EPC**	0.00	0.45	0.87	1.31	1.65	1.99	2.29	2.59	2.91	3.11	3.39
**Average C**	0.00	0.43	0.85	1.18	1.53	1.79	2.05	2.26	2.47	2.63	2.81
**ΔR**	0.00	0.01	0.01	0.13	0.11	0.19	0.23	0.32	0.44	0.48	0.57

Condition 1. Reproductive success for simulated roving focal male in Condition 1 at pair population 500 at each percentage of roving by other males in the population.

Pair Population 500	0%	10%	20%	30%	40%	50%	60%	70%	80%	90%	100%
**Average Total R**	4.00	3.98	4.00	4.10	4.10	4.21	4.23	4.35	4.45	4.49	4.57
**Average IPC**	4.00	3.54	3.13	2.80	2.48	2.20	1.91	1.73	1.56	1.36	1.20
**Average EPC**	0.00	0.44	0.87	1.30	1.62	2.01	2.32	2.62	2.89	3.13	3.37
**Average C**	0.00	0.45	0.86	1.19	1.52	1.80	2.08	2.26	2.44	2.63	2.79
**ΔR**	0.00	-0.01	0.01	0.10	0.10	0.21	0.23	0.35	0.45	0.49	0.57

Condition 2.

Reproductive success for simulated roving focal male in Condition 2 at pair population 1000 with 20% wandering males at each percentage of roving by other males in the population.

Pair Population 1000 with 20% Wanderers	0%	10%	20%	30%	40%	50%	60%	70%	80%	90%	100%
**Average Total R**	4.00	3.25	3.28	3.37	3.46	3.59	3.68	3.83	3.88	4.00	4.15
**Average IPC**	4.00	2.83	2.47	2.20	1.94	1.72	1.54	1.36	1.18	1.05	0.95
**Average EPC**	0.00	0.42	0.81	1.17	1.52	1.87	2.14	2.47	2.70	2.95	3.20
**Average C**	0.00	1.17	1.53	1.80	2.05	2.27	2.46	2.63	2.81	2.95	3.04
**ΔR**	0.00	-0.74	-0.71	-0.62	-0.53	-0.40	-0.31	-0.16	-0.11	0.01	0.15

Condition 2.

Reproductive success for simulated roving focal male in Condition 2 at pair population 1000 with 30% wandering males at each percentage of roving by other males in the population.

Pair Population 1000 with 30% Wanderers	0%	10%	20%	30%	40%	50%	60%	70%	80%	90%	100%
**Average Total R**	4.00	2.87	2.97	3.13	3.18	3.32	3.45	3.59	3.69	3.84	3.94
**Average IPC**	4.00	2.47	2.19	1.97	1.73	1.52	1.36	1.22	1.07	0.96	0.83
**Average EPC**	0.00	0.40	0.78	1.16	1.45	1.80	2.09	2.37	2.62	2.88	3.11
**Average C**	0.00	1.53	1.81	2.03	2.26	2.48	2.64	2.77	2.93	3.03	3.16
**ΔR**	0.00	-1.12	-1.02	-0.86	-0.81	-0.67	-0.54	-0.40	-0.30	-0.15	-0.05

Condition 3. Reproductive success for simulated roving focal male in Condition 3 at pair population 1000 with 20% wandering males and a balanced sex-ratio from unpaired females at each percentage of roving by other males in the population.

**Pair Population 1000 with 20% Wanderers**	**0%**	**10%**	**20%**	**30%**	**40%**	**50%**	**60%**	**70%**	**80%**	**90%**	**100%**
**Average Total R**	4.00	4.05	4.08	4.22	4.26	4.32	4.39	4.48	4.58	4.71	4.78
**Average IPC**	4.00	2.81	2.46	2.21	1.93	1.71	1.52	1.35	1.19	1.07	0.96
**Average EPC**	0.00	1.24	1.62	2.01	2.33	2.61	2.87	3.13	3.39	3.64	3.82
**Average C**	0.00	1.19	1.53	1.78	2.06	2.28	2.48	2.64	2.81	2.93	3.04
**ΔR**	0.00	0.05	0.08	0.22	0.26	0.32	0.39	0.48	0.58	0.71	0.78

Condition 3. Reproductive success for simulated roving focal male in Condition 3 at pair population 1000 with 30% wandering males and a balanced sex-ratio from unpaired females at each percentage of roving by other males in the population.

**Pair Population 1000 with 30% Wanderers**	**0%**	**10%**	**20%**	**30%**	**40%**	**50%**	**60%**	**70%**	**80%**	**90%**	**100%**
**Average Total R**	4.00	4.15	4.19	4.24	4.31	4.38	4.44	4.57	4.69	4.81	4.88
**Average IPC**	4.00	2.50	2.21	1.94	1.71	1.52	1.32	1.19	1.06	0.96	0.84
**Average EPC**	0.00	1.65	1.98	2.30	2.60	2.86	3.12	3.38	3.63	3.85	4.04
**Average C**	0.00	1.50	1.79	2.05	2.28	2.47	2.67	2.80	2.94	3.03	3.16
**ΔR**	0.00	0.15	0.19	0.24	0.31	0.38	0.44	0.57	0.69	0.81	0.88

Abbreviations:	
**Average Total R**	Average reproductive success achieved via extra-pair and intra-pair copulations
**Average IPC**	Average reproductive success achieved via intra-pair copulations
**Average EPC**	Average reproductive success achieved via extra-pair copulations
**Average C**	Average reproductive success lost via cucolding
**ΔR**	Total change in reproductive success
